# Sexual dimorphism in the relationship between brain complexity, volume and general intelligence *(g)*: a cross-cohort study

**DOI:** 10.1038/s41598-022-15208-4

**Published:** 2022-06-30

**Authors:** Anca-Larisa Sandu, Gordon D. Waiter, Roger T. Staff, Nafeesa Nazlee, Tina Habota, Chris J. McNeil, Dorota Chapko, Justin H. Williams, Caroline H. D. Fall, Giriraj R. Chandak, Shailesh Pene, Murali Krishna, Andrew M. McIntosh, Heather C. Whalley, Kalyanaraman Kumaran, Ghattu V. Krishnaveni, Alison D. Murray

**Affiliations:** 1 Aberdeen Biomedical Imaging Centre, School of Medicine, Medical Sciences and Nutrition, University of Aberdeen, Lilian Sutton Building, Foresterhill, Aberdeen, AB25 2ZD UK; 2 Aberdeen Royal Infirmary, NHS Grampian, Aberdeen, UK; 3 School of Public Health, Imperial College London, London, UK; 4 Gold Coast University Hospital, Southport, QLD Australia; 5 MRC Lifecourse Epidemiology Unit, University of Southampton, Southampton, UK; 6 Genomic Research on Complex Diseases, CSIR - Centre for Cellular and Molecular Biology, Hyderabad, India; 7 Department of Imaging and Interventional Radiology, Narayana Multispecialty Hospital, Mysore, India; 8 Foundation for Research and Advocacy in Mental Health, Mysore, India; 9 Division of Psychiatry, Centre for Clinical Brain Sciences, Royal Edinburgh Hospital, University of Edinburgh, Edinburgh, UK; 10 Epidemiology Research Unit, CSI Holdsworth Memorial Hospital, Mysore, India

**Keywords:** Cognitive neuroscience, Image processing

## Abstract

Changes in brain morphology have been reported during development, ageing and in relation to different pathologies. Brain morphology described by the shape complexity of gyri and sulci can be captured and quantified using fractal dimension (FD). This measure of brain structural complexity, as well as brain volume, are associated with intelligence, but less is known about the sexual dimorphism of these relationships. In this paper, sex differences in the relationship between brain structural complexity and general intelligence (*g*) in two diverse geographic and cultural populations (UK and Indian) are investigated. 3D T1-weighted magnetic resonance imaging (MRI) data and a battery of cognitive tests were acquired from participants belonging to three different cohorts: Mysore Parthenon Cohort (MPC); Aberdeen Children of the 1950s (ACONF) and UK Biobank. We computed MRI derived structural brain complexity and *g* estimated from a battery of cognitive tests for each group. Brain complexity and volume were both positively corelated with intelligence, with the correlations being significant in women but not always in men. This relationship is seen across populations of differing ages and geographical locations and improves understanding of neurobiological sex-differences.

## Introduction

The human brain has a complex structure with cortical folding defining gyri and sulci. Cortical folding develops in utero and continues as the brain grows into late adolescence^1^ under the influence of both genetic and mechanical factors, with rapid cell growth in the cortical plate driving expansion and axonal tension driving gyrification^2^. The shape of the brain and its complexity can be quantified using a topological measure, fractal dimension (FD), which captures the shape properties at different scales using fractal geometry^3–5^. The higher the details and irregularities of the cortical sheet, a more rapid degree of self-similar scaling, the higher the FD. During normal brain development, FD increases until adolescence^1,6^ and then decreases through adulthood to late life^7–11^. Brain volume has a similar trajectory across the life span^12^.

The FD of cortical folding has been studied in pathologic conditions^13^, where differences in patients with schizophrenia^14–16^, bipolar disorder^17^, Alzheimer’s disease^18^, multiple sclerosis^19^, epilepsy^20^, intellectual disabilities^21^, autism^22^, dyslexia^23^, asymptomatic carotid stenosis^24^ compared to healthy controls have been found.

On the other hand, there are known brain differences between men and women^25,26^. While men generally have larger brain volumes and surface areas compared with women, women have higher cortical thickness^25^ and higher structural complexity (FD) in two specific brain regions, the superior-frontal and parietal lobes^27^. A better understanding of brain morphology and cognitive differences between men and women can provide insights into brain-related ailments which differ by sex. For example, rates of Alzheimer’s disease are higher in women than men^28^, Major Depressive Disorder most frequently affects women and more of them become treatment-resistant than their male counterparts^29^, while men are more frequently affected by schizophrenia^14^, autism spectrum disorders^30^ and dyslexia^23^.

Most research shows that larger brain volume is associated with higher intelligence^31^ and that brain atrophy is a significant marker of brain ageing^7^. Cox et al.^32^ found no difference between the sexes in the association between total brain volume and general intelligence *(g)*. Structural brain complexity (FD) is positively associated with intelligence^33,34^, long term cognitive development^35^ and cognitive change over the life course^36^ and duration of education^33^. However, there is a scarcity of evidence on the sex differences in the relationship of brain complexity and intelligence, given the knowledge that men and women have differing average brain volumes.

Here, we test the hypotheses that greater brain complexity and volume are associated with greater general intelligence *(g)* and the strength of this association is greater in women, using data from Indian and UK cohorts.

## Results

All the reported results are focused on brain complexity, brain volumes and general intelligence *g* for the Indian cohort (Mysore Parthenon Cohort, MPC) and two UK cohorts (Aberdeen Children of the 1950s, ACONF and UK Biobank) with respect to sex. Age was considered in the model for UK Biobank because the age range was larger (45-79 y).

### Brain complexity

The complexity of brain shape described by gyri and sulci was quantified using FD. The values of FD for the whole brain with respect to sex and cohorts are reported in Table 1. Whole brain complexity is significantly greater for men than women in all cohorts.

**Table 1:** Differences in whole brain complexity (FD) between sexes for all data sets: the Indian sample (Mysore Parthenon Cohort, MPC), Scottish (Aberdeen Children of the 1950s, ACONF) and UK Biobank.

Cohorts (age years)	Complexity: males, mean ± sd	Complexity: females, mean ± sd	Complexity: males (min; max)	Complexity: females (min; max)	*t*	*p*
MPC (20–22)	2.6041 ± .0112	2.5910 ± .0120	2.5794; 2.6331	2.5632; 2.6132	t(164) = − 7.256	< .001
ACONF (60–66)	2.6344 ± .0104	2.6226 ± .0104	2.6051; 2.6563	2.5960; 2.6523	t(236) = − 8.75	< .001
UKBiobank (60–66)	2.6382 ± .0098	2.6292 ± .0093	2.6047; 2.6696	2.5961; 2.6695	t(1967) = − 19.11	< .001
UKBiobank (45–79)	2.6381 ± .0102	2.6300 ± .0097	2.5976; 2.6728	2.5871; 2.6695	t(6657) = − 33	< .001

### Brain volume

The values for whole brain volumes (cm^3^) without ventricles with respect to sex and cohorts are reported in Table 2. Brain volume is significantly greater for men than women in all cohorts.

**Table 2:** Differences in brain volume between sexes for all data sets: the Indian sample (Mysore Parthenon Cohort, MPC), Scottish (Aberdeen Children of the 1950s, ACONF) and UK Biobank.

Cohorts (age years)	Brain Volume (cm^3^): males, mean ± sd	Brain Volume (cm^3^) females, mean ± sd	*t*	*p*
MPC (20–22)	1126.58 ± 88.86	998.43 ± 84.62	t(164) = − 9.50	< .001
ACONF (60–66)	1122.28 ± 92.05	1002.09 ± 84.35	t(236) = − 10.51	< .001
UK Biobank (60–66)	1157.58 ± 92.00	1073.02 ± 80.24	t(1967) = − 28.88	< .001
UK Biobank (45–79)	1187.42 ± 97.616	1072.75 ± 82.96	t(6657) = − 51.80	< .001

### General intelligence *g*

Principal components analysis was used to identify the first unrotated principal component of the combined cognitive tests, Table 3. The component loadings of each cognitive test are also given in the table.

**Table 3:** The first unrotated component and how much it accounts for the variance in the scores for all subjects and the component loadings.

Cohorts	The variance explained by general factor *g* (first component)/the component loadings		
	All subjects	Females	Males
MPC	52.63% (N = 166)	55.21% (N = 80)	51.74% (N = 86)
	Block design: 0.714	Block design: 0.780	Block design: 0.636
	Digit span: 0.807	Digit span: 0.796	Digit span: 0.817
	Matrix reasoning: 0.807	Matrix reasoning: 0.844	Matrix reasoning: 0.770
	Arithmetic: 0.796	Arithmetic: 0.845	Arithmetic: 0.773
	Symbol search: 0.551	Symbol search: 0.449	Symbol search: 0.659
	Visual Puzzle: 0.728	Visual Puzzle: 0.756	Visual Puzzle: 0.706
	Information: 0.672	Information: 0.701	Information: 0.684
	Coding: 0.693	Coding: 0.695	Coding: 0.690
ACONF	84.67% (N = 238)	82.37% (N = 122)	86.85% (N = 116)
Childhood	Verbal test 1: 0.938	Verbal test 1: 0.920	Verbal test 1: 0.954
	Verbal test 2: 0.936	Verbal test 2: 0.926	Verbal test 2: 0.945
	Arithmetic test: 0.901	Arithmetic test: 0.891	Arithmetic test: 0.911
	English test: 0.904	English test: 0.893	English test: 0.916
ACONF	42.52% (N = 238)	43.66% (N = 122)	41.58% (N = 116)
Adult	Logical memory immediate recall: 0.810	Logical memory immediate—recall: 0.791	Logical memory immediate recall: 0.817
	Logical memory delayed recall: 0.800	Logical memory delayed recall: 0.759	Logical memory delayed recall: 0.828
	Digit symbol: 0.401	Digit symbol: 0.432	Digit symbol: 0.348
	Verbal Fluency: 0.604	Verbal Fluency: 0.619	Verbal Fluency: 0.581
	Mill Hill Vocabulary: 0.681	Mill Hill Vocabulary: 0.709	Mill Hill Vocabulary: 0.676
	Matrix reasoning: 0.516	Matrix reasoning: 0.587	Matrix reasoning: 0.477
UK Biobank	44.01% (N = 6659)	43.39% (N = 3505)	44.01% (N = 3154)
	Log Reaction Time: − 0.554	Log Reaction Time: 0.556	Log Reaction Time: − 0.568
	Verbal-numeric Reasoning: 0.616	Verbal-numeric Reasoning: − 0.603	Verbal-numeric Reasoning: 0.606
	Log of no of incorrect pairs matches: − 0.560	Log of no of incorrect pairs matches: 0.572	Log of no of incorrect pairs matches: − 0.557

The principal component was transformed into an IQ-like score, general intelligence *g* with a mean of 100 and a standard deviation of 15. Values for all groups and ages are shown in Table 4. *g* was significantly higher for men than women only in UK Biobank data, contrary to what is found in the ACONF cohort where there was a trend for *g* computed at ages 60–66 y to be higher for women, but no significant difference in other groups.

**Table 4:** Differences in general intelligence *g* between sexes for all data sets: the Indian sample (MPC), Scottish (ACONF) and UK Biobank.

Cohorts	Age at cognitive testing (years)	General intelligence *g*: males, mean ± sd	General intelligence *g*: females, mean ± sd	General intelligence *g:* Males (min; max)	General intelligence *g*: females (min; max)	*t*	*p*
MPC	20–22	98.62 ± 14.52	101.48 ± 15.45	64.40; 131.18	64.07; 136.85	t(164) = − 1.23	.220
ACONF	11	98.76 ± 15.59	101.18 ± 14.38	59.35; 128.91	66.97; 131.37	t(236) = − 1.25	.213
ACONF	60–66	98.10 ± 14.84	101.81 ± 14.98	57.48; 133.47	65.78; 132.72	t(236) = − 1.91	.057
UKBiobank	60–66	102.27 ± 16.39	99.40 ± 15.75	47.32; 141.98	56.95; 142.64	t(1967) = − 3.96	< .001
UKBiobank	45–79	101.09 ± 16.77	99.02 ± 15.87	47.32; 142.53	51.24; 143.17	t(6657) = − 5.17	< .001

### Correlations between brain complexity and general intelligence *g* and their comparisons

The relationships between whole brain complexity determined from magnetic resonance images (MRI) and intelligence with respect to sex differences in two different geographic and cultural populations (UK and Indian) were investigated.

**Figure 1:**
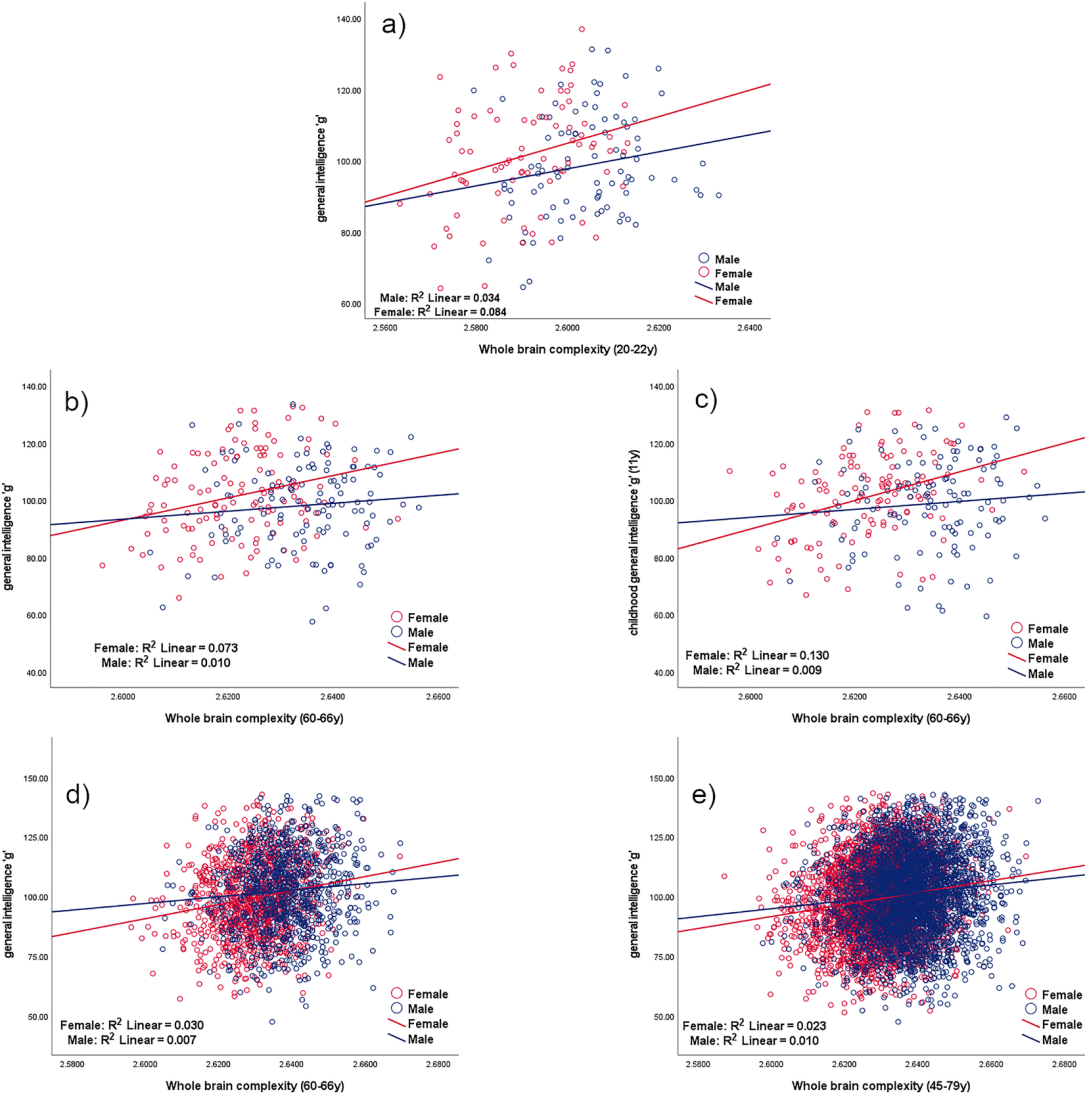
Correlations between whole brain complexity and general intelligence *g* according to sex. (**a**) Mysore Parthenon Cohort (MPC)—correlation between whole brain complexity and general intelligence *g* for women (red) and men (blue) at age 20–22. (**b**,**c**) ACONF cohort—correlation between whole brain complexity and general intelligence *g* at age 60–66 (**b**) and at age 11 (**c**) for women (red) and men (blue). (**d**,**e**) UK Biobank—correlation between whole brain complexity and general intelligence *g* at age 60–66 (**d**) and at age 45–79 (**e**) for women (red) and men (blue).

The main results are shown in Table 5 and Fig. 1 and show Pearson correlations between brain complexity for whole brain and general intelligence *g*. There are significant correlations in women between brain complexity and general intelligence *g* for all groups, but there is no significant correlation for men in the first two cohorts. There are significant differences between the two correlations corresponding to men and women and also in their corresponding slopes in UK Biobank and just for childhood *g* and brain complexity in ACONF.

**Table 5:** Pearson correlations between whole brain complexity and general intelligence *g* and correlation comparison between sexes; where *r* is the correlation coefficient; **correlation is significant at the .01 level (2-tailed), *correlation is significant at .05 level (2-tailed), the probability *p* < .05 is uncorrected; test statistic *z* for correlation comparison (First transformed the r to Z using the Fisher Z transformations so that they were normally distributed and then applied a Z test where the differences in the Z measured was divided by the standard error. A Z score of greater than ± 1.96 was considered significant), and *t* statistic for the slope difference.

Correlation between general intelligence *g* and whole brain complexity	All	Women	Men	Correlation comparison (Women and Men)	Slope comparison (Women and Men)
**Mysore Parthenon Cohort** Correlation between general intelligence *g* and brain complexity at age 20–22	r = .160* ***p* = .040** **N = 166**	r = .291** ***p* = .009** **N = 80**	r = .184 *p* = .089 N = 86	z = .718 *p* = .237	t = .682 *p* = .495
**ACONF** Correlation between childhood general intelligence *g*^a^ and brain complexity at age 60–66	r = .154* ***p* = .017** **N = 238**	r = .361** ***p* < .001** **N = 122**	r = .094 *p* = .318 N = 116	**z = 2.16** ***p* = .015**	**t = 3.027** ***p* = .003**
**ACONF** Correlation between general intelligence *g* at age 60–66 and brain complexity at the same age (60–66)	r = .099 *p* = .127 N = 238	r = .270** ***p* = .003** **N = 122**	r = .098 *p* = .296 N = 116	z = 1.36 *p* = .086	t = 1.898 *p* = .059
**UK Biobank** Correlation between general intelligence *g* and brain complexity at age 60–66^b^	r = .154** ***p* < .001** **N = 1969**	r = .174** ***p* < .001** **N = 1062**	r = .083* ***p* = .012** **N = 907**	**z = 2.045** ***p* = .021**	**t = 2.060** ***p* = .040**
**UK Biobank** Correlation between general intelligence *g* and brain complexity at age 45–79	r = .141** ***p* < .001** **N = 6659**	r = .153** ***p* < .001** **N = 3505**	r = .101** ***p* < .001** **N = 3154**	**z = 2.153** ***p* = .016**	**t = 2.120** ***p* = .034**

The significant values are shown in bold.

^a^Childhood general intelligence *g* data are not contemporaneous with MRI acquisition as for the rest of data.

^b^ The UK Biobank group 60–66 y was retrieved from the UK Biobank data (45–79 y) in order to match the ACONF age group for a better comparison with an increased number of participants.

### Correlations between brain volume and general intelligence *g* and their comparisons

The associations between brain volume determined from magnetic resonance images (MRI) using FreeSurfer software and intelligence with respect to sex differences in these cohorts were investigated similarly as for the brain complexity.

**Figure 2:**
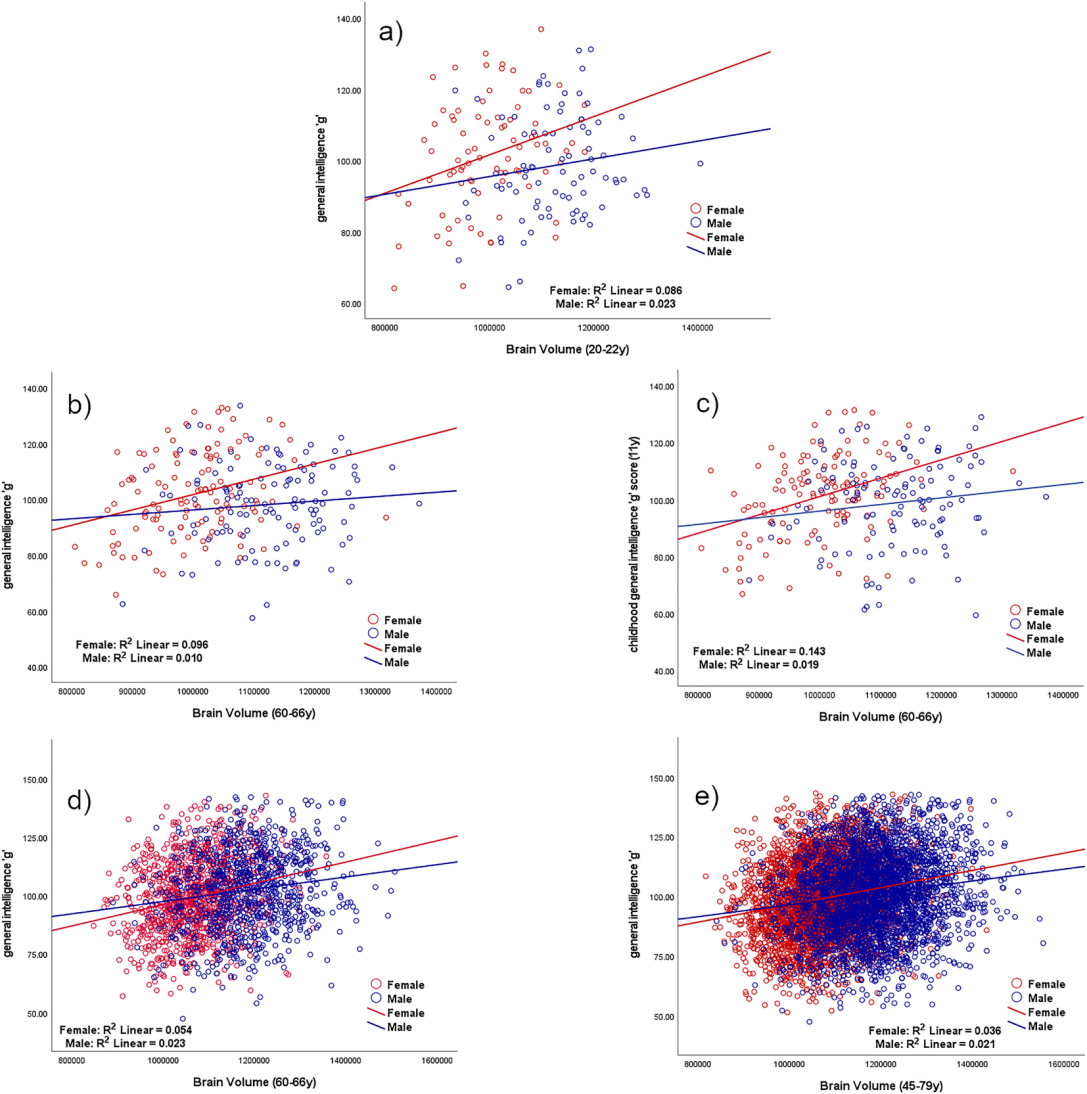
Correlations between brain volume (mm^3^) and general intelligence *g* according to sex. (**a**) Mysore Parthenon Cohort (MPC)—correlation between brain volume (mm^3^) and general intelligence *g* for women (red) and men (blue) at age 20–22. (**b**,**c**) ACONF cohort—correlation between brain volume (mm^3^) and general intelligence *g* at age 60–66 (**b**) and at age 11 (**c**) for women (red) and men (blue). (**d**,**e**) UK Biobank—correlation between brain volume (mm^3^) and general intelligence *g* at age 60–66 years (**d**) and at age 45–79 years (**e**) for women (red) and men (blue).

The main results are shown in Table 6 and Fig. 2 and show the correlations between brain volume and general intelligence *g*. There are significant correlations in women between brain volume and general intelligence *g* for all groups, but there is no significant correlation for men in the first two cohorts. There are significant differences between the two correlations corresponding to men and women and also in their corresponding slopes in ACONF and UK Biobank.

**Table 6:** Pearson correlations between brain volume and general intelligence *g* and correlation comparison between sexes; where *r* is the correlation coefficient; **correlation is significant at the .01 level (2-tailed), *correlation is significant at .05 level (2-tailed), the probability *p* < .05 is uncorrected; test statistic *z* for correlation comparison (First transformed the r to Z using the Fisher Z transformations so that they were normally distributed and then applied a Z test where the differences in the Z measured was divided by the standard error. A Z score of greater than ± 1.96 was considered significant), and *t* statistic for the slope difference.

Correlation between general intelligence *g* and brain volume	All	Women	Men	Correlation comparison (Women and Men)	Slope comparison (Women and Men)
**Mysore Parthenon Cohort** Correlation between general intelligence *g* and brain volume at age 20–22	r = .119 *p* = .125 N = 166	r = .294** ***p* = .008** **N = 80**	r = .152 *p* = .161 N = 86	z = .94 *p* = .174	t = 1.088 *p* = .278
**ACONF** Correlation between childhood general intelligence *g*^a^ and brain volume at age 60–66	r = .160* ***p* = .013** **N = 238**	r = .378** ***p* < .001** **N = 122**	r = .137 *p* = .141 N = 116	**z = 1.98** ***p* = .024**	**t = 3.634** ***p* < .001**
**ACONF** Correlation between general intelligence *g* at age 60–66 and brain volume at the same age (60–66)	r = .095 *p* = .142 N = 238	r = .309** ***p* < .001** **N = 122**	r = .098 *p* = .295 N = 116	**z = 1.68** ***p* = .046**	t = 1.816 *p* = .071
**UK Biobank** Correlation between general intelligence *g* and brain volume at age 60–66^b^	r = .207** ***p* < .001** **N = 1969**	r = .232** ***p* < .001** **N = 1062**	r = .151* ***p* < .001** **N = 907**	**z = 1.86** ***p* = .**032	**t = 2.346** ***p* = .019**
**UK Biobank** Correlation between general intelligence *g* and brain volume at age 45–79	r = .173** ***p* < .001** **N = 6659**	r = .189** ***p* < .001** **N = 3505**	r = .145** ***p* < .001** **N = 3154**	**z = 1.84** ***p* = .033**	**t = 2.560** ***p* = .010**

The significant values are shown in bold.

^a^Childhood general intelligence *g* data are not contemporaneous with MRI acquisition as for the rest of data.

^b^The UK Biobank group 60–66 y was retrieved from the UK Biobank data (45–79 y) in order to match the ACONF age group for a better comparison with an increased number of participants.

### Interaction sex*brain complexity (FD) in all cohorts

For MPC, the two-way ANOVA shows a main effect of brain complexity (*p* = 0.002), but not a sex*brain complexity interaction F(1,162) = 0.466, *p* = 0.496, partial ɳ^2^ = 0.003, observed power = 0.104. In the ACONF cohort when childhood general intelligence g is the dependent variable; sex and complexity have significant main effects and the interaction sex*brain complexity is also significant (*p* = 0.048) in the model F(1,234) = 3.935, *p* = 0.048, partial ɳ^2^ = 0.017 and estimated power = 0.506. In the same ACONF cohort but when the adult general intelligence *g* is the dependent variable, the main effect is significant for brain complexity (*p* = 0.004), but not for sex*brain complexity interactions (F(1,234) = 1.875, *p* = 0.172, partial ɳ^2^ = 0.008 and estimated power = 0.276).

Conducting a similar analysis in a retrieved subgroup of UK Biobank, which matched for age those from ACONF, we found main effects of sex (*p* = 0.039), brain complexity (*p* < 0.001) and sex*brain complexity interaction (*p* = 0.039) in the model F(1,1965) = 4.265, *p* = 0.039, partial ɳ^2^ = 0.002 and estimated power = 0.541.

The whole UK Biobank sample, which has a large age range (45-79y), was analysed using the same model as previously, but adding age as a covariate due to the large range. We found main effects of sex (*p* = 0.012), brain complexity (*p* < 0.001), age (*p* < 0.001) and a sex*brain complexity interaction (*p* = 0.013) in the model F(1,6654) = 6.238, *p* = 0.013, partial ɳ^2^ = 0.001 and estimated power = 0.704.

### Differences in ${R}^{2}$ across groups and sexes

${R}^{2}$ as a measure of fit quality for the slope which provides the value of FD (Fig. 5) was analysed. A two-way ANOVA was conducted that examined the effect of sex and cohorts on $R^{2}$. There was a statistically significant interaction between the effects of sex and groups (cohorts) level on $R^{2}$, *F *(2, 7057) = 12.351, *p* < 0.001. The males have significantly a better fit than females (*p* < 0.001) in each cohort reflected in a bigger $R^{2}$.

### Interaction between brain complexity (FD) and total intracranial volume (TIV)

We tested the interaction of FD*TIV in all three groups. For MPC the model shows a main effect of sex (*p* = 0.022), FD main effect (*p* = 0.055) as a trend, and no interaction FD*TIV F(1,161) = 2.388, *p* = 0.122, partial ɳ^2^ = 0.015, observed power = 0.336. In the ACONF cohort when adult general intelligence *g* is the dependent variable; sex, FD and TIV have significant main effects and the FD*TIV interaction is also significant in the model F(1,233) = 4.994, *p* = 0.026, partial ɳ^2^ = 0.021 and estimated power = 0.605.

For the whole UK Biobank sample, we added, as mentioned previously, age as a covariate due to the large age range (45-79 y) and we found main effects of sex, FD, TIV, age and FD*TIV interaction: F(1,6653) = 8.037, *p* = 0.005, partial ɳ^2^ = 0.001 and estimated power = 0.809.

However when the sex variable was removed from the above models to see if the FD*TIV interactions persist we found significance for ACONF: F(1,234) = 4.651, *p* = 0.032, ɳ^2^ = 0.019 and estimated power = 0.575; and for UK Biobank: F(1,6654) = 8.250, *p* = 0.004, ɳ^2^ = 0.001 and estimated power = 0.819.

### Association between brain complexity, brain volume and age in UK Biobank

**Figure 3:**
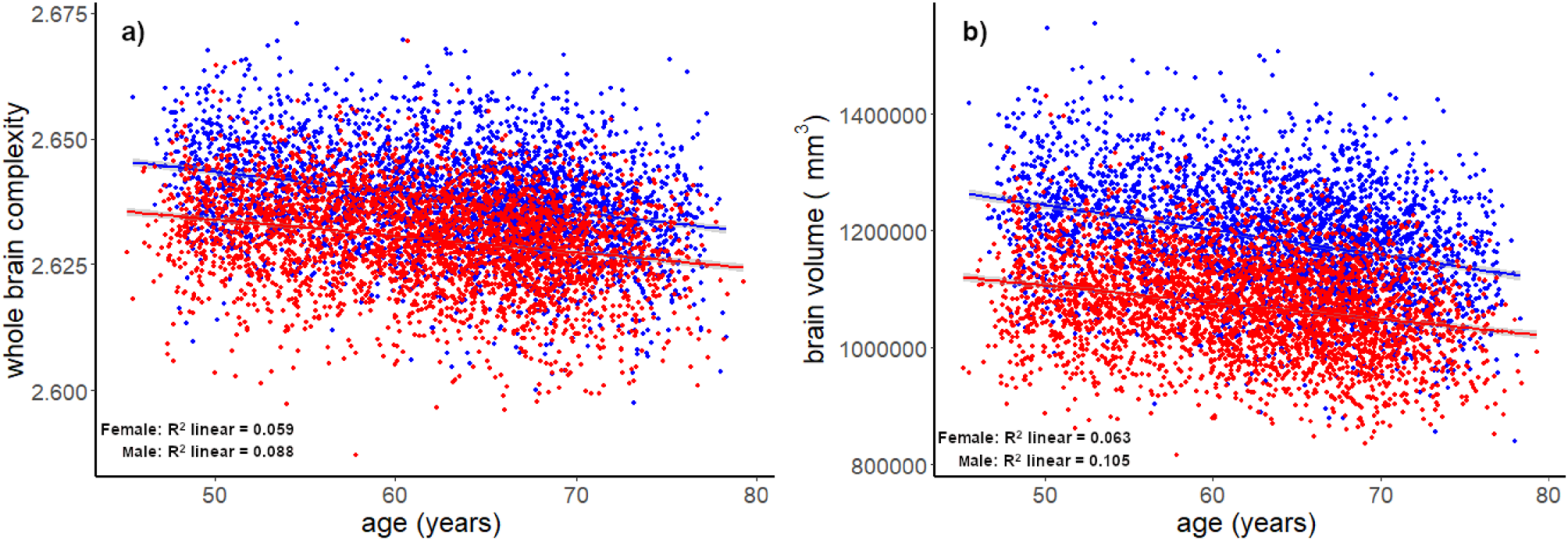
UK Biobank—correlation between whole brain complexity (**a**), volume mm^3^ (**b**) and age (45–79 y) for women (red) and men (blue).

We analysed the correlation between the structural brain complexity (FD), volume and age. As expected, there was a decline of complexity and volume with age (Fig. 3) and a negative correlation between complexity and age for men (r = − 0.296; *p* < 0.001; N = 3154) and women (r = − 0.242; *p* < 0.001; N = 3505), similarly for brain volume and age for men (r = − 0.324; *p* < 0.001; N = 3154) and women (r = –0.248; *p* < 0.001; N = 3505).

### Comparions for cortical complexity and general inteligence *g* in different geographical groups

Considering the decline of brain complexity with age, as proved previously in the manuscript, an investigation was carried out between the youngest cohort (MPC) and ACONF, where the cognitive tests belong to similar bateries (WAIS). The comparison was done for each sex separatly. A significant diference was noticed for the brain complexity between the Indian and UK group with a higher complexity in ACONF cohort, despite elderly age in ACONF (Table 7). No significant difference was noticed for general inteligenge *g* between these two cohorts (Table 7).

**Table 7:** Sex-differences in complexity (FD) and general inteligence *g* between MPC (Indian cohort) and ACONF (an UK cohort); with *t* statistic and the probability *p* < *.05,* mentioned in the table.

Cohort (N)	Males (mean ± sd)				Females (mean ± sd)			
	MPC (N = 86)	ACONF (N = 116)	t	*p*	MPC (N = 80)	ACONF (N = 122)	t	*p*
FD	2.6041 ± .0112	2.6344 ± .0104	t(200) = − 19.76	< .001	2.5910 ± .0120	2.6226 ± .0104	t(200) = − 19.86	< .001
*g*	98.62 ± 14.52	98.10 ± 15.59	t(200) = .248	.804	101.48 ± 15.45	101.81 ± 14.38	t(200) = − 1.47	.883

## Discussion

This study shows that across different age and cultural groups the associations between both shape complexity, as measured by FD, and volume of the human brain and intelligence are significant for women, however, this is not always true for men (Table 5, Table 6). When we compare these associations between sexes, significant differences are found for UK Biobank (all data and 60–66 y group), for ACONF comparisons of the correlations between childhood *g* and FD and also for ACONF where there is a tendency for the correlations between contemporaneous *g* collected together with MRI data and FD. This significance and tendency are found not just for comparisons between correlations but also for the slope difference, which consolidate the findings.

The interaction sex*brain complexity exists in the cohorts and occasions as those where significance was found in sex-difference between comparisons of correlations in Table 5.

More than this, in Table 6, in addition to the similar significance as in Table 5, a new significant sex-difference of the correlation between brain volume and *g* appears for ACONF where *g* is derived from data contemporaneous with MRI collection.

Bearing all of this in mind, the trend towards sex-differences in the associations between *g* and FD was found to be consistent across cohorts and age. When we compare the sex-differences in the correlation between *g* and brain volume the trend is maintained, which underlies the idea that there is sexual dimorphism in the association between brain morphology and intelligence*.*

Another finding is a better fit reflected in a greater $R^{2}$ of the slope, which defines the FD value in a double logarithmic plot of number of boxes versus box size that needed to cover the brain, for men than women across all groups. It is hard to interpret this as an error considering that the correlation between brain volume and *g* creates or tends to create the same sexual dimorphism, rather it can be interpreted as a slightly different topologic organisation of the brain between sexes.

A more rapid degree of self-similar scaling of the brain is associated with a higher intelligence for women. The association between brain complexity and higher intelligence in women than in men might be explained as an adaptation to accommodate a large cortical surface area (brain) in a small volume (skull). However, considering the significant interaction between FD and total intracranial volume (TIV) in ACONF and UK Biobank, in models with sex included as a variable and also without, it is still hard to conclude that it is sex-specific rather than a sex-independent principle of brain organisation. These findings may explain why individuals with smaller intracranial volume, but higher cortical complexity, have the same intelligence as individuals with a bigger intracranial volume. Females versus their male counterparts might be a good example.

Even if it wasn’t the main purpose of this study, the correlation between brain complexity, volume and age was sought in the UK Biobank where this was possible due to the large range of age, 45–79. This analysis was generated rather for replicability of other studies and confirmation that the brain complexity computed in this manuscript follows the same pattern with age (Fig. 3). It is already known that the brain complexity and volume decline with age^7^.

Looking at other secondary findings, there is no difference between general intelligence *g* in different geographical groups (MPC and ACONF) analysing each sex separately, but there is a difference in brain structural complexity between these groups (Table 7). The groups in this comparison are of different age. Negligible sex differences in *g* were found in another article which used Primary Mental Abilities (PMA) battery^37^. The difference in complexity might be explained through spatial mismatches and mislocalizations between Indian and Caucasian brains and there is also a significant difference in size, with the Indian brain being smaller on average in terms of length, width, and height^38^. There are morphological differences in the brain by ethnicity and described by human phenotype as shown in other populations (Chinese, African Americans, Japanese)^39–41^.

Matching the UK biobank data for *g* in both sex groups using a case matching approach (Online Appendix A) shows similar effects, with the differences between the sex being of greater significance. One of the criticisms (limitations) of our work is the fact that we have not corrected for multiple comparisons. However, these could be mostly considered complementary rather than repeat measures because of the marked differences between populations the samples are drawn from, with the exception of the two UK Biobank samples. The MPC is drawn from a south-east Asian population situated in a developing nation, and the ACONF sample is drawn from a population in the north-east of Scotland that has experienced a remarkable economic transformation due to an oil boom throughout their working lives, making changes to their socioeconomic position, life experience and opportunity. The UK Biobank data is drawn from across the UK and is more representative of the UK population than the others. As discussed, the small size of the MPC and ACONF samples may be underpowered. However, the direction of the differences supports our UK Biobank findings. In addition, the matched case–control analysis shown in Online Appendix A would survive any multiple comparison correction for the two UK Biobank samples.

Posthuma et al.^42^ reported correlations between grey matter and white matter volume and *g* of r = 0.25 and r = 0.24, respectively, and it is likely that these correlations are of genetic origin^43^. On the other hand, Cox et al.^32^, looking at the association between *g* and total brain volume in the UK Biobank participants, did not find sex differences. However, Cox used Structural Equation Modelling (SEM) to investigate this relation and different cognitive tests than used here. Nave et al.^31^ found no interaction between sex and total brain volume influences on fluid intelligence, despite a positive correlation between fluid intelligence and *g*. Our results suggest a potential sexual dimorphism in the relationship between brain structural complexity and intelligence, and brain volume and intelligence; stressing that the brain shape complexity and brain volume support each other in these findings.

Whole brain complexity in women predicts cognitive ability (Fig. 1). This relationship is true even for childhood intelligence (Fig. 1c), and thus brain structural complexity can be considered a conservation of distinct genetically mediated human cortical patterns^44^ and as a consequence a biomarker of cognitive resilience for use in epidemiological studies. Prenatal conditions have an influence on brain complexity^45^ and also environmental factors such as paternal education and maternal ethnicity also intervene during pregnancy on cortex development^46^ and early childhood environmental factors might have their impact too, which we plan to investigate in future studies. Schmitt et al. found no associations between cortical complexity and polygenic risk for either schizophrenia, bipolar disorder or psychiatric cross-disorder, driving the conclusion that potential environmental risk factors during pregnancy play an important role^47^. These risk factors during pregnancy such as smoking, maternal age, pre-pregnancy body mass index, and use of acetaminophen are associated with maternal risk alleles^48^. Brain complexity is likely to be sensitive to genetic heritage, prenatal and early postnatal brain development as well as later changes throughout the life span^9,16,17,47^. Structural complexity decline with age was found in the UK Biobank cohort, where the participants were from middle to late adulthood (45–79 y), these findings are supported by literature^7–11^.

One of the limitations of this study is that the age is not matched for all groups. The Indian cohort was younger (20–22 y) compared with the other cohorts: ACONF (60–66 y) and UK Biobank (45–79 y). For ACONF and UK Biobank matched groups were created and analysed; and for the models containing all UK Biobank data, age was included as a covariate. It is also important to note that general intelligence *g* was derived from different cognitive tests for each cohort, which may introduce cognitive domain biases in *g*. Related to this, the sample size was considerably bigger for UK Biobank, which allows much smaller effect sizes to be identified. Another potential limitation could be that collecting MRI data across different scanner manufacturers might include slight variation among scans.

Despite the sample size differences between cohorts, the large number of participants from UK Biobank makes this analysis one of the largest datasets where FD, as a measure of brain structural complexity has been applied.

Neurobiological sex-differences can provide a clue in understanding neurodevelopmental and neurodegenerative aspects which can evolve differently in function of sex.

This paper builds on the sexual dimorphism of cortical complexity introduced by Luders et al.^27^ in a sample of 60 participants. Here we demonstrate that the relationship between both structural brain complexity, brain volume and cognitive ability tend to be stronger in women than in men and seem to be consistent across populations of different ages and geographical locations.

## Methods

### Participants

The participants belong to three human population cohorts with geographical and cultural differences in the UK and India.

We computed magnetic resonance imaging (MRI) derived structural brain complexity from an Indian cohort (age 20–22 y), and two cohorts in the UK: one from Scotland (60–66 y) and the other from the UK Biobank (45–79 y). Included in this study are 166 participants (86 males) from the Mysore Parthenon Cohort (MPC)^49^, from Mysore, South India; 238 participants (122 males) from the Aberdeen Children of the 1950s (ACONF) cohort, Scotland; and 6659 participants (3154 males) from the January 2017 data realise of UK Biobank. The data from participants were collected: in India through a collaborative pilot project; those from Scotland as part of Generation Scotland^50^; and from UK Biobank^51^. The datasets are contemporary, with collection starting in 2014. The Scotland and India data collection finished in 2016 and 2019 respectively, UK Biobank data collection is ongoing.

All participants provided written informed consent prior to the collection of any data or samples for all three cohorts: MPC, ACONF and UK Biobank. All research was performed in accordance with the relevant local as well as international guidelines. The MPC study was approved by the institutional ethics committee of CSI Holdsworth Memorial Hospital, Mysore, which is constituted as per the guidelines of the Indian Council of Medical Research. For the ACONF cohort, ethical approval for the study was obtained from the Scotland A Research Ethics Committee (REC reference number 14/55/0039) and the local Research and Development offices. UK Biobank received ethical approval from the North West Multi-Centre Research Ethics Committee (11/NW/03,820). The research using the UK Biobank Resource was conducted under Application Number 24089 (PI Waiter).

### Cognitive data

The participants have contemporaneous cognitive data from a battery of culturally validated tests administered at the time of acquisition of MRI. In addition, the ACONF participants have also age 11 cognitive ability measures. The tests administrated in Mysore are WAIS-IV ^(India)^ and contain 8 subtests (Block Design, Digit Span, Matrix Reasoning, Arithmetic, Symbol Search, Visual Puzzle, Information, Coding), which measure crystallised and fluid intelligence, and short- and long-term memory. Four cognitive tests were collected in Aberdeen at age 11, as part of the Aberdeen Child Development Survey (Verbal reasoning T1, Verbal reasoning T2, English test, Arithmetic)^52^. There were six other cognitive tests collected in Aberdeen at age 60–66, which are validated and widely used cognitive tests that measure crystallised- and fluid-type cognitive tasks (Verbal fluency, Mill Hill Vocabulary, Logical memory—immediate recall, Logical memory—delayed recall, Digit symbol and Matrix reasoning), including a United Kingdom version of the Logical Memory subtest from the Wechsler Memory Scale-III (WAIS-III).

UK Biobank participants were administered tests that measured fluid intelligence (http://biobank.ctsu.ox.ac.uk/crystal/field.cgi?id=20016): reaction time, verbal-numeric reasoning, and visual memory (no of incorrect pairs matches). The format and content of cognitive task were partly novel^53^.

### General intelligence *g*

General intelligence *g* is a concept which is used in research into the individual differences in general human intelligence. It proposes that an individual’s general intelligence underlies their ability in multiple different cognitive tests and helps understanding the neurological mechanism behind them^54^.

Principal component analysis (PCA) was used for data reduction^55,56^: the first unrotated principal component *g* from the cognitive tests collected from each battery of cognitive tests on each occasion in Mysore, in Aberdeen and in UK Biobank data, respectively. Before computing *g* in UK Biobank, log transforms were used for two tests: reaction time and visual memory errors but not for verbal-numerical reasoning^53^. PCA also allows to identify how much variance is shared between tests. An IQ-like score, named general intelligence *g,* was computed through standardizing the first unrotated principal component *g* multiplying by 15 and adding 100.

### MRI data acquisition

The characteristics of the MRI data are detailed in the Table 8:

**Table 8:** MRI data characteristics.

Location	Manufacturer	Field strength	Sequence	Matrix	Tr (ms)	Te (ms)	Resolution (mm)
Aberdeen	Philips	3 T	FGRE	256 × 256x160	2124	3.8	0.94 × 0.94 × 1
Mysore	GE	1.5 T	FSPGR	256 × 256x160	2934	4.97	0.94 × 0.94 × 1
UK Biobank	Siemens	3 T	MPRAGE	256 × 256x205	2000	4.0	1 × 1 × 1

Whole brain MRI volumes and total intracranial volume (TIV) have been extracted using the processing in FreeSurfer 6.0 (https://surfer.nmr.mgh.harvard.edu/).

### Brain complexity

Complexity and self-organisation are everywhere in nature from the level of microorganisms such as bacteria^57^ upwards. The term “complexity” as used in neuroscience is very broad and covers many different topics from the shape of neurons to brain topology to biological signals^5,13^ and networks^58,59^. In this article complexity refers to the degree of self-similar scaling of brain shape and was captured using the box-counting method.

**Figure 4:**
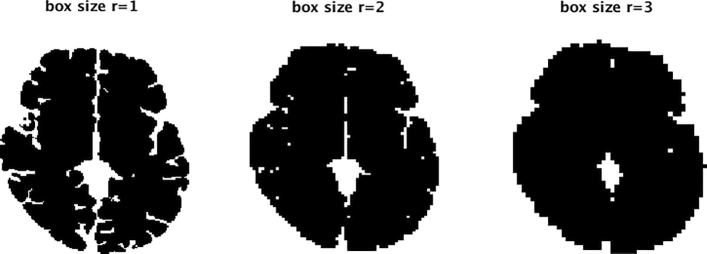
A two-dimensional illustration of the box-counting method for a trans axial slice, which is covered with boxes of increasing size. The section is extracted after the construction of boxes on three-dimensional brain mask.

**Figure 5:**
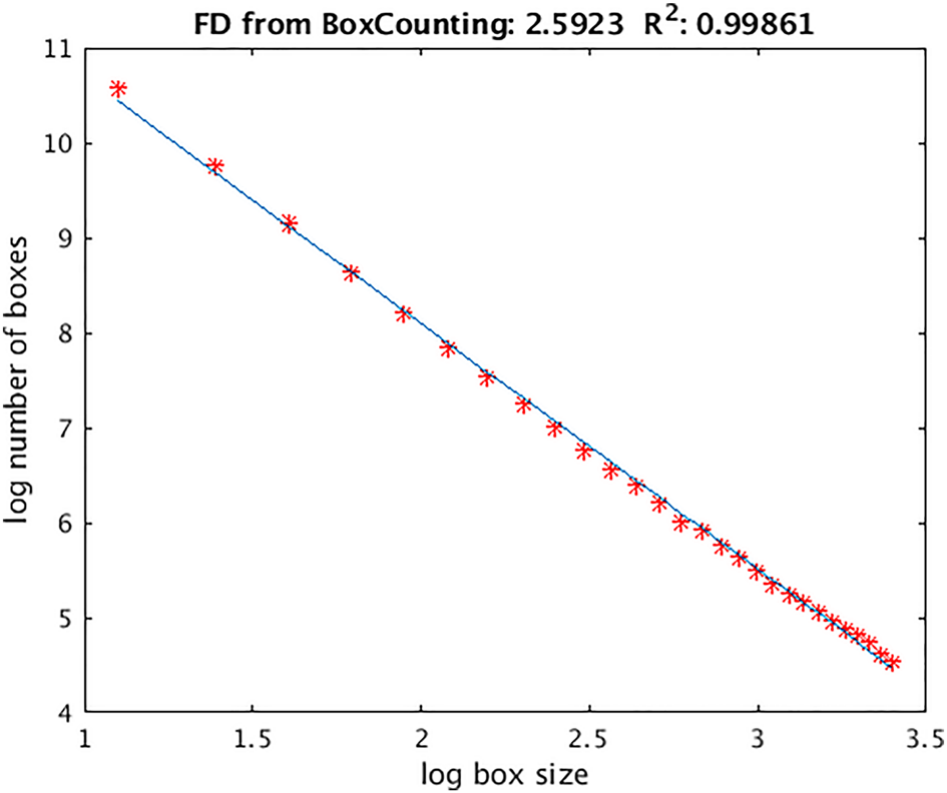
Logarithmic plot of the number of boxes containing the brain mask versus box size. The fractal dimension given by the slope is 2.5923, R^2^ = 0.99861.

The input image for the calculation of this type of complexity is a binary brain mask extracted using FreeSurfer 6.0 (https://surfer.nmr.mgh.harvard.edu/) for each individual. Brain complexity was measured using fractal dimension (FD) and computed using the box-counting method applied to the whole brain mask using an in-house written software in Matlab^9,15^. The process is exemplified in Fig. 4, where an axial slice from one of the subjects participating in this study is shown, covered with boxes of increasing size. A box was counted as a “hit” if at least one voxel of the brain was located within the box. The number of boxes (*N*) of a given length needed to cover the whole brain structure varies with the linear size (*r*) of the box as *N* ~ *r*^*−D*^, where *D* is the fractal dimension given by the slope in a double logarithmic plot of number of boxes versus box size. The scaling is linear. For whole brain structure ***r*** is iterated within the range from r = 3 to r = 30 voxels. A representative example of the slope is given in Fig. 5 for the same participant as for the box-counting method illustration, with the calculated slope and R^2^ included, showing the quality of the fit. The validation procedure of the method was done using digital phantoms with a known fractal dimension^15^. The reliability of the FD measurements has been established^15^ and has been shown to be higher than other cortical metrics such as cortical thickness^60^.

### Statistics

Statistical analyses were conducted with SPSS version 27 and R software. For groups comparisons independent samples t-tests were used. Pearson (bivariate) correlations between whole brain complexity, volume and general intelligence *g* were performed across all participants and for both sexes individually. Using the Fisher r-to-z transformation, the significance of the difference between these two correlation coefficients between the sexes within each cohort was assessed. The difference between slopes described by correlations was computed using *t*-statistics.

Based on previous literature, there is ample evidence to assume that there is a linear relationship between brain volume, complexity and intelligence^33,35,36^. We have followed this assumption and have applied General Linear Modelling methods. Using a univariate General Linear Model, with general intelligence *g* considered the dependent variable, brain complexity as the independent variable with age as a covariate, where the age range is large; and sex as a fixed factor we tested interaction of sex*brain complexity. A two-way ANOVA was used to evaluate whether there was a significant interaction between sex and whole brain complexity. Another similar model was designed using also a univariate General Linear Model to test the interaction of brain complexity*total intracranial volume (FD*TIV). The differences in R^2^ as a measure of fit quality for the slope which provides the value of FD across groups and sexes were tested using a two-way ANOVA model.

## Supplementary Information

Supplementary Information.
