# Supplementary material for: Sexual dimorphism in the relationship between brain complexity, volume and general intelligence (g): a cross-cohort study
Source: Sci Rep. 2022 Jun 30;12:11025. doi: 10.1038/s41598-022-15208-4 (PMC9247090; doi:10.1038/s41598-022-15208-4)
Supplement: Supplementary file 1 — Supplementary Information. [file 41598_2022_15208_MOESM1_ESM.docx]

Appendix A

One of the weaknesses of our primary analysis was the differences in *g* values between sexes when using the UK Biobank data. The result is unexpected and contrary to multiple findings in the literature. This appendix re-exams the UK Biobank data using a matched case-control approach, ensuring that *g* is similar for both sexes.

Matched groups were using the case-controlled analysis^1^ approach in SPSS. A pair was said to be matched if their *g* values were within 1 point.

The matching process identified 876 participants for both sexes for the UK Biobank 60-66y group discarding 11% of the original data. The matching process identified 3063 participants in the complete UK Biobank data set, discarding 8% of the data. Estimates of the complexity (FD), volume and *g* for both sexes are shown in Tables 1A-3A.

Table 4A shows the correlations between *g* and FD, and Table 5A shows the correlations between *g* and BV for each sex. The statistical differences between these correlations and slopes were compared using the approach outlined in the main manuscript.

| Cohorts  (age years) | Complexity:  males,  mean ± sd | Complexity: females,  mean ± sd | Complexity:  males  (min; max) | Complexity:  females  (min; max) | *T* | *P* |
| --- | --- | --- | --- | --- | --- | --- |
| UKBiobank(60-66) | 2.6381 ± .0097 | 2.6301 ± .0093 | 2.6047; 2.6696 | 2.5961; 2.6695 | t(1750)=-17.47 | <.001 |
| UKBiobank(45-79) | 2.6380 ± .0102 | 2.6302 ± .0096 | 2.5976; 2.6728 | 2.5871; 2.6695 | t(6124)=-31.09 | <.001 |

Table 1A. Differences in whole-brain complexity (FD) between sexes in UK Biobank Data Sets

| Cohorts  (age years) | Brain Volume ($cm^{3}$):  males,  mean ± sd | Brain Volume ($cm^{3}$ )  females,  mean ± sd | *T* | *P* |
| --- | --- | --- | --- | --- |
| UK Biobank(60-66) | 1156.49 ± 91.44 | 1048.32 ± 79.18 | t(1750)=-26.47 | <.001 |
| UK Biobank(45-79) | 1159.10± 95.04 | 1047.80 ± 80.78 | t(6124)=-49.38 | <.001 |

Table 2A. Differences in brain volume between sexes in UK Biobank data sets

| Cohorts | Age at cognitive testing  (years) | General intelligence *g*: males,  mean ± sd | General intelligence *g*: females,  mean ± sd | General intelligence *g:*  males  (min; max) | General intelligence *g*:  females  (min; max) | *t* | *P* |
| --- | --- | --- | --- | --- | --- | --- | --- |
| UKBiobank | 60-66 | 101.51 ± 15.66 | 101.44 ± 15.62 | 56.56; 141.98 | 58.03; 142.64 | t(1750)=-.093 | .926 |
| UKBiobank | 45-79 | 100.32 ± 16.12 | 100.26 ± 16.08 | 52.03; 142.53 | 51.24; 143.17 | t(6124)=-.124 | .901 |

Table 3A. Differences in general intelligence *g* between sexes in UK Biobank

| **Correlation between general intelligence *g* and whole brain complexity** | **All** | **Women** | **Men** | **Correlation comparison (Women and Men)** | **Slope**  **comparison**  **(Women and**  **Men)** |
| --- | --- | --- | --- | --- | --- |
| **UK Biobank**  Correlation between general intelligence *g* and brain complexity at age 60-66 | r =.111**  p < .001  N = 1752 | r= .178**  p < .001  N = 876 | r= .063  p = .063  N = 876 | z = 2.44  p = .007 | t = 2.54  p = .011 |
| **UK Biobank**  Correlation between general intelligence *g* and brain complexity at age 45-79 | r = .114**  p < .001  N = 6126 | r = .150**  p < .001  N = 3063 | r= .097**  p < .001  N = 3063 | z = 2.106  p = .018 | t = 2.38  p = .017 |

Table 4A. Pearson correlations between whole-brain complexity and general intelligence *g* and correlation comparison between sexes; where *r* is the correlation coefficient; **correlation is significant at the .01 level (2-tailed), *correlation is significant at .05 level (2-tailed), the probability p*<*.05 is uncorrected; test statistic *z* for correlation comparison and *t* statistic for the slope difference. The significant values are shown in red.

| **Correlation between general intelligence *g* and brain volume** | **All** | **Women** | **Men** | **Correlation comparison (Women and Men)** | **Slope**  **comparison**  **(Women and**  **Men)** |
| --- | --- | --- | --- | --- | --- |
| **UK Biobank**  Correlation between general intelligence *g* and brain volume at age 60-66 | r = .155**  p < .001  N = 1752 | r = .241**  p < .001  N = 876 | r= .132**  p < .001  N = 876 | z = 2.36  p = .009 | t = 2.87  p = .004 |
| **UK Biobank**  Correlation between general intelligence *g* and brain volume at age 45-79 | r = .136**  p < .001  N = 6126 | r = .187**  p < .001  N = 3063 | r = .138**  p < .001  N = 3063 | z = 1.97  p = .024 | t = 2.96  p = .003 |

Table 5A. Pearson correlations between brain volume and general intelligence *g* and correlation comparison between sexes; where *r* is the correlation coefficient; **correlation is significant at the .01 level (2-tailed), *correlation is significant at .05 level (2-tailed), the probability p*<*.05 is uncorrected; test statistic *z* for correlation comparison and *t* statistic for the slope difference. The significant values are shown in red.

References:

1. Bruce N.G., Pope D., Stanistreet D.L. in *Quantitative Methods for Health Research: A Practical Interactive Guide to Epidemiology and Statistics*. (2018 John Wiley & Sons, Ltd, 2018), Second Edition 2017, pages 257-305.
